# Beyond the Surface: A Case Report of Verrucous Carcinoma in a Young Female

**DOI:** 10.7759/cureus.70367

**Published:** 2024-09-28

**Authors:** Neha Sethi, Manjusha Agrawal, Archan Patel, Anubha Dande, Lucky Srivani Reddy

**Affiliations:** 1 Obstetrics and Gynaecology, Jawaharlal Nehru Medical College, Datta Meghe Institute of Higher Education and Research, Wardha, IND

**Keywords:** myocutaneous flap, radical vulvectomy, rare malignancy, squamous cell carcinoma, verrucous carcinoma, vulvar tumour

## Abstract

Verrucous carcinoma is a rare, low-grade variant of squamous cell carcinoma, characterised by slow growth and local invasiveness. While typically observed in older individuals, it can also present in younger patients, posing diagnostic challenges. A 30-year-old female presented to the obstetrics and gynaecology department with a three-month history of a foul-smelling, ulcerative perineal mass measuring 15 × 10 cm. Histopathological analysis confirmed the diagnosis of verrucous carcinoma. The patient had significant anaemia and required transfusion therapy prior to surgery. A radical vulvectomy with reconstruction using a myocutaneous flap was performed. The surgical approach involved wide local excision with a 1 cm margin around the tumour, followed by defect reconstruction. Postoperative recovery was uneventful, and histopathological examination confirmed complete excision with clear surgical margins. Verrucous carcinoma of the vulva is rare, particularly in young females, and early recognition is crucial for effective management. This case underscores the importance of accurate diagnosis and timely surgical intervention to ensure optimal outcomes. A multidisciplinary approach and comprehensive care were essential for successful treatment and recovery. Given the tumour’s potential for local recurrence, careful long-term follow-up is recommended.

## Introduction

Verrucous carcinoma (VC) is a rare, well-differentiated variant of squamous cell carcinoma (SCC) that exhibits distinct clinical and pathological features, making its diagnosis and treatment a challenge. Initially described by Ackerman in 1948, VC is known for its slow, exophytic growth and locally invasive behaviour but with a notably low potential for metastasis [1]. While VC can affect various anatomical regions, such as the oral cavity (often called Ackerman's tumour), the larynx, and the anogenital areas, its occurrence in the vulva is exceptionally rare, particularly in younger women [2]. Vulvar cancers are themselves uncommon, with SCC accounting for approximately 90% of cases [3]. However, VC represents less than 1% of these cases, underscoring its rarity in this anatomical region [4]. The pathogenesis of VC remains poorly understood, though several factors have been associated with its development. Chronic irritation and inflammation, poor hygiene, and human papillomavirus (HPV) infection, particularly with low-risk HPV types like HPV-6 and HPV-11, are commonly implicated in the aetiology of VC in anogenital regions [5]. Nonetheless, HPV infection is not universally present in cases of VC, and a significant proportion of patients may not have any identifiable risk factors, as seen in this case. The absence of high-risk HPV subtypes (HPV-16 and HPV-18), typically associated with more aggressive SCCs, is also a distinguishing feature of VC [6].

Clinically, VC often presents as a slow-growing, wart-like lesion with a verrucous surface. The exophytic nature of the tumour and its well-defined borders make it visually distinct from other malignancies [7]. However, despite its indolent appearance, VC can cause significant local destruction if not diagnosed and treated promptly. In the vulvar region, patients may present with discomfort, pain, discharge, or bleeding as the tumour enlarges and ulcerates, mimicking the appearance of benign lesions like condyloma acuminata [8]. Due to these similarities, VC can be easily misdiagnosed, which may delay appropriate treatment. Histopathologically, VC is characterised by a well-differentiated appearance, with broad, pushing borders and minimal nuclear atypia. Unlike conventional SCC, VC lacks the typical invasive growth pattern that penetrates deeper into the tissue stroma [8]. The tumour grows in a papillomatous or verrucous pattern, which helps differentiate it from more aggressive SCCs. Although VC does not tend to metastasise to lymph nodes or distant organs, its ability to invade adjacent tissues underscores the importance of early diagnosis and complete surgical excision [9].

Surgical resection remains the gold standard treatment for VC, with wide local excision being the most commonly recommended approach [10]. Given the tumour's tendency for local recurrence, clear surgical margins are essential. Radical vulvectomy is often necessary in cases where the lesion is extensive, as in this patient. In some cases, reconstruction using a myocutaneous flap may be required to ensure both functional and aesthetic outcomes [11]. In contrast to other types of SCC, VC responds poorly to radiation therapy and chemotherapy due to its low mitotic activity and slow growth, limiting the utility of non-surgical interventions [12]. Although the prognosis for patients with VC is generally favourable when treated early, local recurrence is a concern, especially if clear margins are not achieved during the initial surgery [12]. Long-term follow-up is recommended to monitor for recurrence, given that VC can recur years after initial treatment [13]. This case underscores the importance of maintaining a high index of suspicion for VC, particularly in patients presenting with chronic vulvar lesions, to facilitate early intervention and improve clinical outcomes.

## Case presentation

**Figure 1:**
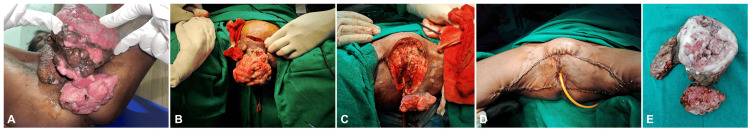
(A-C) A 15 x 10 cm mass originating from the left vulvar region. (D) A postoperative image of the surgical site. (E) The excised mass along with the intervening skin.

A 30-year-old female presented to the obstetrics and gynaecology department at a tertiary care hospital in central India with a chief complaint of a perineal mass that had persisted for three months. Upon initial evaluation, the patient was vitally stable. A local examination revealed a 15 × 10 cm mass originating from the left vulvar region (Figure 1A-C), which was foul-smelling and accompanied by purulent discharge, raising suspicion of malignancy.

**Figure 2:**
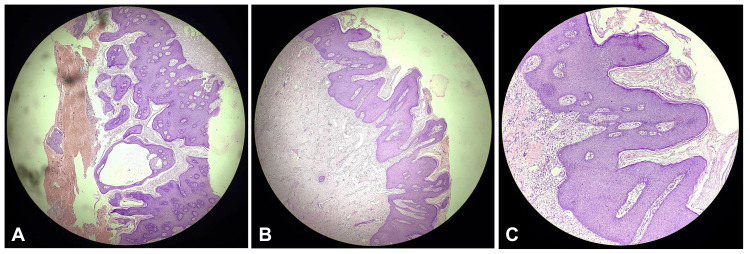
(A-C) Multiple focal biopsies were performed to assess the nature of the mass. Histopathological examination of the samples confirmed a diagnosis of verrucous carcinoma, a rare, low-grade variant of squamous cell carcinoma. This type of carcinoma is characterized by slow growth and local invasiveness, with a low likelihood of metastasis.

Multiple focal biopsies were taken to determine the nature of the mass. Histopathological analysis of the biopsy samples confirmed VC, a rare, low-grade variant of SCC known for its slow growth and local invasiveness but low potential for metastasis (Figure 2). The patient was also found to have significant anaemia and was transfused with four units of packed red blood cells. Once she was haemodynamically stable, a surgical plan was devised. The patient subsequently underwent a radical vulvectomy with reconstruction using a myocutaneous flap.

The surgical approach involved marking an incision around the mass. The outer incision encompassed the labia majora, including the clitoris and anterior half of the perineal skin, ensuring a 1 cm margin clear of the tumour. A circular inner incision was made around the vaginal introitus, beginning just below the external urethral meatus. The mass, along with the intervening skin, superficial fascia, erectile tissue, and deep fascia, was meticulously dissected and excised, exposing the underlying muscle. Hemostasis was achieved through ligation of the pudendal vessels and vessels supplying the clitoris (Figure 1D-E).

The defect was reconstructed using a myocutaneous flap and a sterile dressing. Postoperatively, the patient’s recovery was steady and uneventful. Histopathological examination of the excised tissue confirmed VC with clear surgical margins, indicating successful tumour removal. This case highlights the importance of considering VC in the differential diagnosis of vulvar masses, even in younger patients. Early and accurate diagnosis, followed by appropriate surgical intervention, is crucial for optimal outcomes. This patient’s case exemplifies the challenges and successful treatment strategies for managing such a rare malignancy, underscoring the need for vigilance and comprehensive care in similar clinical scenarios.

## Discussion

VC is a rare, low-grade variant of SCC, first described by Ackerman in 1948 as an exophytic, locally invasive tumour with a slow rate of progression and minimal metastatic potential [1]. It typically arises in regions such as the oral cavity, larynx, and genitourinary tract, with the vulva being an uncommon site of occurrence, particularly in young women [14]. The case presented here is notable due to the patient’s age and the mass's rapid progression over three months, necessitating swift medical and surgical intervention. The pathogenesis of VC is thought to be associated with chronic inflammation and HPV infection, particularly HPV types 6 and 11 [15]. However, in many cases, HPV DNA is not detected, suggesting that other aetiological factors such as tobacco use, poor hygiene, or chronic irritation may contribute to its development [16]. Our patient did not report any history of HPV infection or associated risk factors, making her case particularly unusual. This highlights the necessity of maintaining a broad differential diagnosis when evaluating vulvar masses, even in the absence of traditional risk factors.

The clinical presentation of VC is often non-specific. As seen in this case, it may mimic other vulvar pathologies such as infections, benign tumours, or other forms of SCC. The tumour’s exophytic growth and foul-smelling discharge raised initial concerns for a malignant process, which was confirmed through histopathological analysis. Histologically, VC is characterised by well-differentiated keratinocytes with minimal atypia, pushing margins, and a lack of significant mitotic activity [17]. These features differentiate it from more aggressive SCCs, which are more likely to metastasise and show cellular dysplasia. Radical surgery remains the mainstay of treatment for vulvar VC due to its locally invasive nature and resistance to radiotherapy [8]. In this case, a radical vulvectomy with myocutaneous flap reconstruction was performed, achieving clear margins and an excellent postoperative outcome. Previous literature suggests that wide local excision with negative margins is crucial to prevent recurrence, as incomplete excision may lead to local recurrence in up to 70% of cases [6]. Adjuvant radiotherapy is generally avoided in VC due to concerns that it may induce anaplastic transformation and increase the risk of metastatic disease [18].

While most cases of VC are slow-growing, some reports describe cases with more aggressive clinical courses, particularly in younger patients [19]. In our patient, the rapid growth of the tumour over three months and its large size at presentation required prompt surgical intervention to prevent further local invasion. Fortunately, the surgical management was successful, and postoperative histopathological examination confirmed complete excision with clear margins, reducing the likelihood of recurrence. The prognosis for patients with VC is generally favourable, with long-term survival rates approaching 90% following complete excision [6]. However, given the risk of local recurrence, long-term follow-up is essential, particularly in cases where surgical margins are close or unclear [20]. Our patient will continue to undergo regular follow-up visits to monitor for any signs of recurrence. This case underscores the importance of early recognition and diagnosis of VC, particularly in atypical populations such as younger women. Although rare, this condition must be considered in the differential diagnosis of vulvar masses. Surgical excision with clear margins is crucial for optimal outcomes, and ongoing vigilance in postoperative surveillance is essential to prevent recurrence.

## Conclusions

In conclusion, VC of the vulva is a rare, slow-growing, yet locally invasive malignancy that poses diagnostic and therapeutic challenges, particularly in younger patients. This case highlights the importance of considering VC in the differential diagnosis of vulvar masses, even in the absence of traditional risk factors such as HPV infection. Early diagnosis through histopathological confirmation and prompt surgical intervention with clear margins are critical to ensuring favourable outcomes and reducing the risk of recurrence. The successful management of this patient through radical vulvectomy and myocutaneous flap reconstruction underscores the necessity of a multidisciplinary approach in handling such rare malignancies. Long-term follow-up is essential to monitor for any signs of recurrence, ensuring the best possible prognosis for patients affected by this uncommon yet significant disease.
